# Hypoxia-driven metabolic reprogramming of adipocytes fuels cancer cell proliferation

**DOI:** 10.3389/fendo.2022.989523

**Published:** 2022-10-18

**Authors:** R. Aird, J. Wills, K. F. Roby, C. Bénézech, R. H. Stimson, M. Wabitsch, J. W. Pollard, A. Finch, Z. Michailidou

**Affiliations:** ^1^ University/British Heart Foundation (BHF) Centre for Cardiovascular Science, Edinburgh University, Edinburgh, United Kingdom; ^2^ MRC Institute of Genetics and Molecular Medicine, Edinburgh University, Edinburgh, United Kingdom; ^3^ University of Kansas Medical Center, Kansas City, Kansas, KS, United States; ^4^ University Medical Center Department of Pediatrics and Adolescent Medicine, Ulm, Germany; ^5^ Medical Research Council (MRC) Centre for Reproductive Health, Edinburgh University, Edinburgh, United Kingdom; ^6^ Centre for Tumour Biology, Barts Cancer Institute, Queen Mary University of London, London, United Kingdom

**Keywords:** hypoxia, adipocytes, cancer cells, metabolites, lipids, obesity

## Abstract

**Objective:**

Obesity increases the risk of certain cancers, especially tumours that reside close to adipose tissue (breast and ovarian metastasis in the omentum). The obesogenic and tumour micro-environment share a common pathogenic feature, oxygen deprivation (hypoxia). Here we test how hypoxia changes the metabolome of adipocytes to assist cancer cell growth.

**Methods:**

Human and mouse breast and ovarian cancer cell lines were co-cultured with human and mouse adipocytes respectively under normoxia or hypoxia. Proliferation and lipid uptake in cancer cells were measured by commercial assays. Metabolite changes under normoxia or hypoxia were measured in the media of human adipocytes by targeted LC/MS.

**Results:**

Hypoxic cancer-conditioned media increased lipolysis in both human and mouse adipocytes. This led to increased transfer of lipids to cancer cells and consequent increased proliferation under hypoxia. These effects were dependent on HIF1α expression in adipocytes, as mouse adipocytes lacking HIF1α showed blunted responses under hypoxic conditions. Targeted metabolomics of the human Simpson-Golabi-Behmel syndrome (SGBS) adipocytes media revealed that culture with hypoxic-conditioned media from non-malignant mammary epithelial cells (MCF10A) can alter the adipocyte metabolome and drive proliferation of the non-malignant cells.

**Conclusion:**

Here, we show that hypoxia in the adipose-tumour microenvironment is the driving force of the lipid uptake in both mammary and ovarian cancer cells. Hypoxia can modify the adipocyte metabolome towards accelerated lipolysis, glucose deprivation and reduced ketosis. These metabolic shifts in adipocytes could assist both mammary epithelial and cancer cells to bypass the inhibitory effects of hypoxia on proliferation and thrive.

## Introduction

Obesity and cancer are leading causes of global morbidity and mortality. Obesity is linked to increased risk of breast, gastrointestinal, liver and renal cancer (1, 2). The mechanisms underlying this link are unclear. Adipocytes may modulate the tumour microenvironment through their secretion of bioactive molecules called adipokines (2). However, cancers that grow in adipocyte-rich microenvironments (breast), or tumours that metastasize to fat cell-enriched sites (omentum) such as ovarian cancers may also derive critical metabolic advantages that support their growth (3). Indeed, cancer cells may actively induce metabolic changes in adipocytes resulting in enhanced lipolysis and increased fat utilisation by the cancer cell mitochondria, as demonstrated in mammary, ovarian and melanoma tumours (3–5). Crucially, adipose tissue in obesity (6–8) and tumours (9–11) share a similar pathogenic feature, local hypoxia, driven by vascular rarefaction and formation of leaky vessels. Hypoxia in obesity leads to activation of the hypoxia inducible transcription factor HIF-1α with consequent adipocyte dysfunction and metabolic abnormalities, mainly insulin resistance (12). Hypoxia in cancers is associated with worsened prognosis, increased metastatic potential, chemo-resistance and stemness (11, 13). Similarly, the same adverse outcomes are seen in obese cancer patients (14). Tumour metabolism is altered drastically with hypoxia, shifting towards a lipogenic phenotype with increased lipid uptake and a preference for unsaturated lipids (15–17). The interaction of adipocytes with cancer cells has been well demonstrated (3, 18), however, it is currently unknown whether, or how, hypoxia controls cancer cell re-programming of adipocyte metabolism/function, tumour growth and metastasis. We have shown that adipocyte lipolysis is controlled by hypoxia through the hypoxia inducible transcription factor HIF-1α and its regulatory prolyl-hydroxylases (PHDs; enzymes that target HIF-1α for degradation in normoxia) (19). Here we demonstrate that the hypoxic micro-environment drives the adipocyte-cancer cell metabolic interactions to promote cancer cell proliferation.

## Methods

### Animals

The *Hif1a* conditional allele (20) on a congenic C57BL/6 background, was crossed with the fatty acid binding protein 4 (*Fapb4*)*-Cre* allele (21), (The Jackson Laboratory) to achieve adipose-specific conditional knockout mice. HIF1α^flox^

(stock number, 007561) mice were purchased from the Jackson Laboratories. Genotyping and recombination efficiency PCRs were performed as previously described (19). In all experiments, control littermates (*Hifa* ^flox^; referred as WT) were used for comparisons. C57BL/6JOlaHSD mice were from Envigo. Animals were bred under standard conditions and fed standard chow (product, 801151; Special Diet Services) *ad libitum* unless stated otherwise. In all experiments, female mice (12 weeks old) were used. Animal studies were performed under licensed approval in accordance with the U.K. Home Office Animals (Scientific Procedures) Act, 1986.

### Cell lines

Human SGBS preadipocytes (22) were maintained in DMEM/Ham’s F12 (1:1) medium (Invitrogen, Paisley, UK) containing 10% fetal calf serum (FCS; Invitrogen), 100 U/ml penicillin (Invitrogen), 100 mg/ml streptomycin (Invitrogen), 33 mM biotin and 17 mM pantothenate. To differentiate SGBS cells into adipocytes, near confluent cells were washed three times with PBS and cultured in FCS-free differentiation medium: DMEM/Ham’s F12 (1:1) medium supplemented with 100 U/ml penicillin, 100 mg/ml streptomycin, 33 mM biotin, 17 mM pantothenate, 0.01 mg/ml human transferrin, 20 nM insulin, 100 nM cortisol, 0.2 nM triiodothyronine, 25 nM dexamethasone, 250 μM 3- isobutyl-1-methylxanthine (IBMX) and 2 μM rosiglitazone (Cayman Chemical, Ann Arbor, MI, USA). After 4 days, this medium was replaced with differentiation medium excluding dexamethasone, IBMX and rosiglitazone, which was changed every 3–4 days. At day 15 after induction of differentiation, conditioned media was prepared in DMEM-phenol-free plus 10%-charcoal-stripped FBS after 24h in normoxia (21% O2) or hypoxia (0.5% O2).

The following human and mouse cancer cell lines were used. The human ovarian SKOV3 (ATCC) and breast triple negative MDA-MB-231 (ATCC) were maintained in high-glucose DMEM (D5796, Sigma) + 10% Fetal Bovine Serum (Gibco), Penicillin/Streptomycin 100u/ml/100μg/ml (Gibco). The human breast epithelial non-tumorigenic cells MCF-10A (ATCC) were maintained in DMEM:F12 (Invitrogen), supplemented with 5% horse serum (Invitrogen), 0.5 μg/ml hydrocortisone (Sigma), 20 ng/ml hEGF (Invitrogen), 10 μg/ml insulin (Sigma), 100 ng/ml cholera toxin (Sigma),100 units/ml penicillin and 100 μg/ml streptomycin (Invitrogen).

The mouse ID8 (ovarian) (23) line was maintained in high-glucose DMEM (D5796, Sigma) + 4% Fetal Bovine Serum (Gibco) + Penicillin/Streptomycin 100u/ml/100μg/ml (Gibco) + Insulin-Transferrin- Selenite 5mg/l, 5mg/l, 5μg/l (Gibco, Life Technologies) and the MET-1 (24) derived from the transgenic mouse mammary tumour virus-polyoma middle tumour-antigen MMTV-PyMT) mouse mammary gland carcinoma model of breast cancer, maintained in Low-glucose DMEM (D5546, Sigma) + 5% Fetal Bovine Serum (Gibco) + Penicillin/Streptomycin 100u/ml/100μg/ml (Gibco) + 5ml L-Glutamine (Gibco).

Co-cultures (or CCM donors/recipient cells) were performed in the same species. Human subcutaneous adipocytes with human breast cancer cells; human omental (visceral) adipocytes with human ovarian cancer cells. For mouse, breast cancer cells co-cultured with primary subcutaneous (axillary) adipocytes and ovarian cancer cells with primary visceral (mesenteric) adipocytes.

### Cancer cell conditioned media

2.5x10^5^ cancer cells (SKOV3, MET-1 and ID8) and 5x10^5^ MDA-MB-231 cells were seeded in 6-well plates in the respective normal culture medium, incubated at 37°C/5% CO2 overnight. Culture media was removed, cells were washed with 1 ml cold DPBS and media replaced with 1 ml of phenol-free DMEM (Zen-Bio). Plates were incubated in normoxia (21% O2) or hypoxia (0.5% O2) overnight. CCM was collected and used immediately fresh or frozen at -80°C for later use.

### Human adipose biopsies and conditioned media preparation

Ethical approval (reference number 15/ES/0094) for the collection, storage and subsequent use of human adipose tissue was granted by The Human Tissue (Scotland) Act, 2006 and informed consent was obtained from each participant. Primary human adipocytes were prepared by collagenase digestion, as previously described (19), from biopsies (subcutaneous and visceral) from obese (mean±SEM for body weight: 114±15kg; BMI: 43±5.9 kg/m^2^; age: 49±4yrs) female patients (n=4) undergoing elective surgery for laparoscopic cholecystectomy in the Royal Infirmary of Edinburgh, UK. Briefly, biopsies collected in Krebs-Phosphate buffer, washed with Dulbecco’s phosphate buffer solution (DPBS) (Gibco) and cut into small pieces using a scalpel and further finely chopped in 2mg/ml Collagenase Type I (Worthington)-Krebs- Phosphate solution in a shaking water-bath at 37°C for 1 hour. The digested adipocytes were passed through a 300μm nylon polyester meshwork (300/46) filter and the adipocytes were washed with DPBS and BSA-free fatty acid (Sigma) solution three times. To prepare adipocyte conditioned media (ACM), approximately 2ml of packed adipocyte volume was cultured in four times of its volume of high-glucose phenol-free DMEM with 10% charcoal-stripped FBS (Gibco, Life Technologies), Penicillin/Streptomycin 100u/ml/100μg/ml (Gibco) and incubated at 37°C in normoxia (21% O2) or hypoxia (0.5% O2) overnight. Conditioned media was frozen immediately on dry ice and stored at -80°C.

### Lipolysis assays

Primary adipocytes, 50μl packed volume of adipocytes cultured in 500μl conditioned media in normoxia (21% O2) or hypoxia (0.5% O2). The release of non-esterified fatty acids (WAKO) and glycerol (Sigma) was measured in 30μl of media collected on ice and immediately processed according to the manufacturer’s instructions.

### Proliferation and Fluro-labelled lipid transfer assays

The CyQuant Direct Cell Proliferation Assay (C35011, Thermo Fisher Scientific Company) was used according to manufacturer’s instructions. Briefly, 5x10^3^ cells in 100ml of culture medium were seeded in a 96-well plate and incubated at 37 ^°^C in humidified 5% CO2 overnight to allow to settle down. 24-hours later, relative fluorescent intensity was measured at Day 0 (baseline). Plates either remained in normoxia or hypoxia for another 3 days, culture media or conditioned media was changed daily. The fluorescence absorbance (relative fluorescence units; RFU) was read by an Infinite plate reader (Tecan) with an excitation at 480nm and emission of 535nm at Days 0 (basal RFU) 1, 2 and 3. In lipid transfer experiments adipocytes were incubated with a fluorescent dodecanoic acid analog (QBT Fatty acid uptake assay, Molecular Devices), for 4 h as per manufacturer’s instructions. Briefly, the adipocytes were washed in Hank’s balanced salt solution (HBSS) containing 0.2% fatty-acid free BSA to remove extracellular fatty acids. Cancer cells were incubated with the labelled adipocytes for 24 h. Adipocytes and extracellular fatty acids were washed away with HBSS containing 0.2% fatty-acid free BSA. Confocal images were acquired using Leica SP8 laser scanning confocal microscope. Quantification of Bodipy neutral lipid dye was performed using Image J software and normalized to numbers of nuclei (DAPI) per field.

### Targeted metabolomics

Human fully differentiated SGBS adipocytes were cultured with MCF10A mammary epithelial cells conditioned media (5x10^5^ cells in DMEM-phenol-free with 10% charcoal-stripped FBS) for 24h in normoxia (21% O2) or hypoxia (0.5% O2). SGBS media was collected on ice and frozen (-80°C) for further analysis. As previously described (25), LC-MS was carried out using a 100 mm x 4.6 mm ZIC-pHILIC column (Merck-Millipore) using a Thermo Ultimate, 3000 HPLC in line with a Q Exactive mass spectrometer. A 32 min gradient was developed over the column from 10% buffer A (20 mM ammonium carbonate), 90% buffer B (acetonitrile) to 95% buffer A, 5% buffer B. 10 μl of metabolite extract was applied to the column equilibrated in 5% buffer A, 95% buffer B. Q. Exactive data were acquired with polarity switching and standard ESI source and spectrometer settings were applied (typical scan range 75-1050). Metabolites were identified based upon m/z values and, where available, retention time matching to standards.

### Statistics

Data acquired from cell lines are from 3 independent experiments. Data are presented as mean+/-SEM using GraphPad prism version 8 (GraphPad, San Diego, California, USA) for statistical analysis. A Students t test was used for comparing 2 groups. For comparisons between >2 groups, ANOVA was used with a Šídák’s multiple comparisons test, significance set at p<0.05. For the LC/MS data analysis, 3 biological triplicates were performed and comparisons between normoxia and hypoxia by Students t test.

## Results

### Hypoxic cancer cells induce adipocyte lipolysis

**Figure 1:**
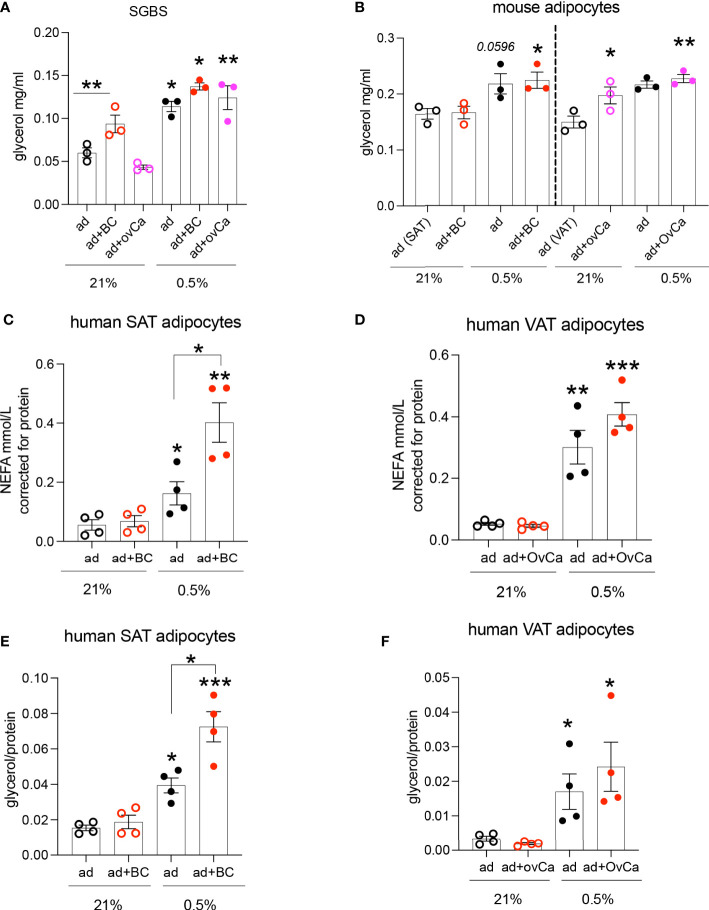
Hypoxic conditioned cancer cell media increases adipocyte lipolysis. **(A)** Glycerol release from SGBS adipocytes cultured in conditioned media (CM) from breast (MDA-MB-231, red) and ovarian (SKOV3, pink) cancer cells respectively under normoxia (21% O2) or hypoxia (0.5% O2), n=3/group (mean from 3 independent experiments). **(B)** Glycerol release from primary C57BL/6J mouse subcutaneous (SAT) and visceral (VAT) adipocytes cultured with CM from MET-1 (red) or ID8 (pink) cancer cells respectively under normoxia (21% O2) or hypoxia (0.5% O2), n=3/group (biological replicates). Non esterified fatty acids (NEFA) **(C, D)** and glycerol **(E, F)** release from human obese subcutaneous (SAT) and visceral (VAT) adipocytes cultured in conditioned media from breast (MDA-MB-231, red; BC) and ovarian (SKOV3, pink; OvCa) cancer cells respectively under normoxia (21% O2) or hypoxia (0.5% O2), n=4/group (biological replicates). Data are mean+/- SEM. Significance by ANOVA *p<0.05, **p<0.01, ***p<0.001. Note: connecting lines show significance within groups in the same O2 tension. If only “*” is used, this indicates significance between different O2 tensions. Abbreviations ad=adipocytes, BC=breast cancer cells, OvCa=ovarian cancer cells. ad+BC (or OvCa) 21%= both cells in 21% O2; ad+BC (or OvCa) 0.5%= both cells in 0.5% O2.

One of the major roles of adipocytes are to store lipids. Co-culture of adipocytes with cancer cells or cancer conditioned media (CCM) has been shown to increase adipocyte lipolysis (3). Given that these cell types will co-exist in a hypoxic microenvironment in the tumour (like breast and ovarian metastasis to omentum), we hypothesized that this hypoxic microenvironment in adipose-associated tumours is a key determinant of alterations in the metabolism of both cell types. We investigated the lipolytic response of the human adipocyte cell line SGBS, C57BL/6J mouse primary adipocytes and human primary adipocytes from obese (BMI: 43±5.9 Kg/m^2^) females, cultured with CCM under hypoxic conditions. CCM was used, instead of co-culture, to minimize the complexity of mixed cells in co-culture and directly assess the CCM effect on adipocyte lipolysis. Hypoxia increased lipolysis in SGBS adipocytes (**Figure 1A**). Although, under normoxia (21% O2), SGBS adipocytes were more sensitive to the lipolytic effects induced by breast compared to ovarian cancer cells (**Figure 1A**), hypoxic (0.5% O2) CCM from both breast and ovarian cells further induced lipolysis in SGBS cells (**Figure 1A**). Similarly, in mouse primary subcutaneous and visceral (mesenteric) adipocytes cultured with CCM prepared from the breast MET-1 and the ovarian ID8 cancer cells respectively, hypoxia had a significantly higher effect on adipocyte lipolysis (**Figure 1B**). In human subcutaneous (SAT) or visceral (VAT) adipocytes from obese subjects, cancer conditioned media under normoxic conditions (21% O2) did not affect the release of non-esterified fatty acids (NEFA) or glycerol by adipocytes (**Figures 1C-F**). Adipocytes in hypoxia (0.5% O2) released more NEFA and glycerol and, when further cultured in a hypoxic-CCM, SAT adipocytes were more sensitive and released more lipids (**Figures 1C, E**) than VAT adipocytes (**Figures 1D, F**). We expected to observe a greater difference in lipolytic rates in visceral adipocytes, however this was not observed in these patients. It is possible that the underlying disease (cholecystitis) of this group may contribute to this discrepancy. The observed differences in glycerol release between human (patient-derived) and mouse adipocytes could be explained by possible inflammation (cholecystitis)-induced lipolysis that could be a potential confounder (26).

### Hypoxic fat cells increase cancer cell proliferation that can be blocked by deleting HIF1α in adipocytes

**Figure 2:**
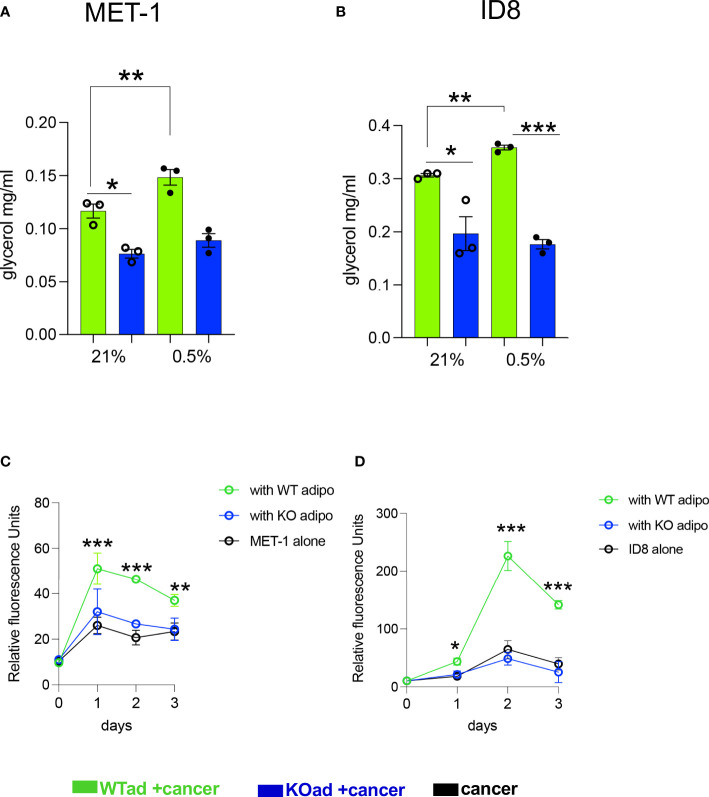
HIF1αKO adipocytes show blunt cancer-induced lipolytic response in hypoxia and do not assist cancer cell proliferation. **(A, B)** Glycerol release from adipose-specific HIF1αKO (KOad, blue bars) or control littermate (WTad, green bars) subcutaneous or visceral fat cells cultured in conditioned media from breast MET-1 or ovarian ID8 cancer cells in normoxia (Nx; 21% O_2_) or hypoxia (Hx;0.5% O_2_, 24h). *In vitro* proliferation of MET-1 **(C)** and ID8 **(D)** cancer cells alone (black) or cultured in adipocyte CM from KO (blue line) or WT (green line) adipocytes for 3 days in hypoxia (0.5% O_2_). Values are mean+/-SEM from 3 independent experiments. * p<0.05, ** p<0.01, ***p<0.001. **(C, D)**, the ACM was prepared in 0.5% O_2._ In the proliferation assay, for MET-1,ACM was prepared from WT or KO SAT adipocytes. For ID-8, the ACM was from VAT adipocytes. p<0.05, ** p<0.01, ***p<0.001. CCM= cancer cell conditioned media; ACM=adipocyte conditioned media.

**Figure 3:**
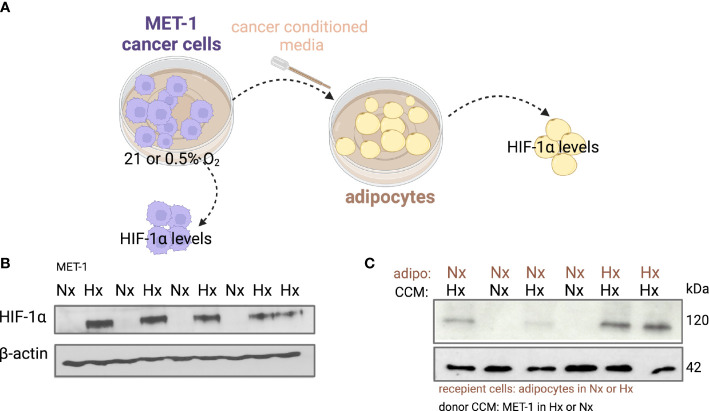
HIF-1α protein levels in adipocytes cultured with hypoxic or normoxic breast cancer MET-1 conditioned media. **(A)** Conditioned media was prepared from MET-1 cells cultured in normoxia (Nx, 21% O_2_) or hypoxia (Hx, 0.5% O_2_), and added to mouse subcutaneous adipocytes cultured in Hx or Nx. Western blot for HIF-1α protein in MET-1 cells **(B)** and adipocytes **(C)** in Nx and Hx. Abbreviations: adipo Nx or Hx=adipocytes cultured in Nx or Hx, CCM Hx = cancer conditioned media prepared in 0.5% O_2_; Nx CCM= cancer conditioned media prepared in 21% O_2_.

Under hypoxic conditions it has been shown that proliferation of cancer cells is significantly reduced or even halted (27, 28). Indeed, when we microscopically checked mouse MET-1 (**Supplemental Figure S1**) and human MDA-MB-231 (**Supplemental Figure S2**) breast cancer cells under hypoxic conditions, their growth and proliferation was significantly reduced. We hypothesised that co-culture with adipocytes would by-pass the inhibitory effect of hypoxia on proliferation and the effect would be HIF-1α dependent. Indeed, under hypoxia, MET-1 and ID-8 cells drove increased lipolysis of wild type (WT) adipocytes, but did not affect lipid release in HIF1α-deficient (KO) adipocytes (**Figures 2A, B**). In turn, mouse MET-1 and ID-8 cells by-passed the inhibitory effect of hypoxia on proliferation when co-cultured with WT mouse primary subcutaneous (**Figure 2C**) and mesenteric adipocytes respectively (**Figures 2D**). In contrast, HIF1α-deficient adipocytes did not assist MET-1 or ID-8 cells to proliferate under hypoxic conditions (**Figures 2C, D**). To further examine whether hypoxic CCM affects HIF1α levels in adipocytes, we first measured HIF1α protein levels in MET-1 cells (**Figure 3A**) and found that are induced in hypoxia (**Figure 3B**). We then prepared CCM from hypoxic (HXCCM) and normoxic (NxCCM) MET-1 and cultured subcutaneous adipocytes in CCM (Figure A). NxCCM had no effect on adipocyte HIF1α levels (**Figure 3C**). Although HIF1α was detected in normoxic adipocytes cultured with HxCCM, this was variable, and lower than HIF1-α expression in hypoxic adipocytes with HxCCM (**Figure 3C**).

### Adipocyte HIF1α deletion limits lipid supply to cancer cells

**Figure 4:**
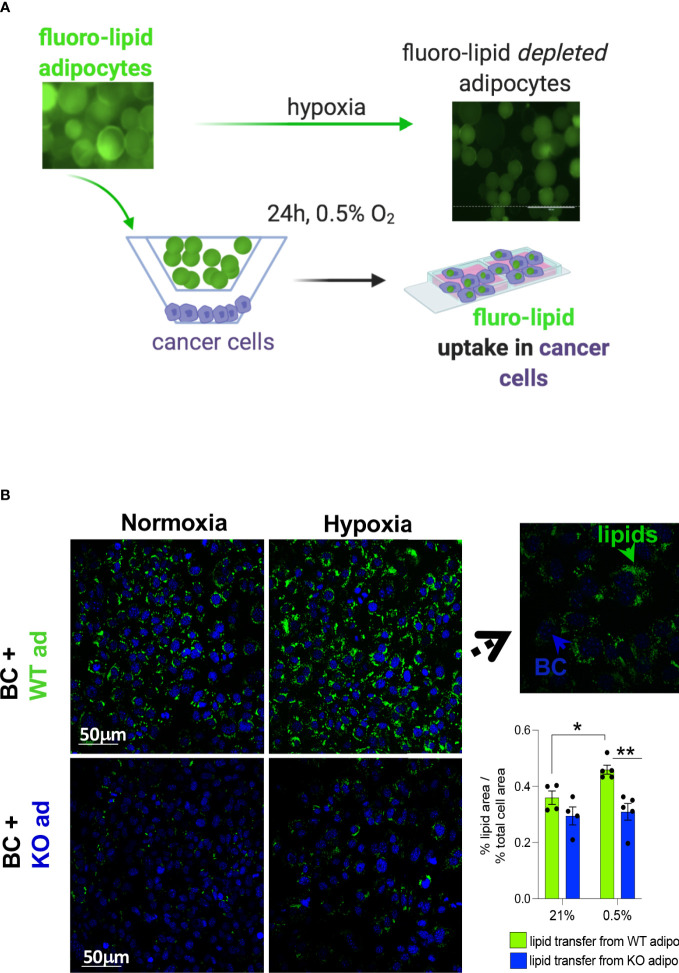
HIF1αKO adipocytes limit lipid transfer to cancer cells in hypoxia. **(A)** mouse primary subcutaneous adipocytes from control littermates (WT) or adipose-specific HIF1αKO (KO) mice were pre-labelled with a fluorescent lipid (fluoro-lipid) analogue. Fluoro-lipid adipocytes were then co-cultured with MET-1 cancer cells for 24h in hypoxia (0.5%) or normoxia (21% O2). Fluro-lipid transfer to MET-1 cells was evaluated by confocal imaging. Note the loss of fluoro-lipid in adipocytes cultured in hypoxia. **(B)** Confocal microscopy images (magnification x40) of fluoro-lipid (green) accumulation in MET-1 (nuclear co-staining, blue) co-cultured with fluoro-labelled HIF1αKO or WT adipocytes. Insert image at x100 magnification indicating that lipids are taken up by MET-1. Fluorescent quantification of % of lipid covered area corrected for the % of area of total nuclei. Scale bars=50 micrometers. Values presented as mean+/- SEM. n=4-5/group. Significance by 2-way ANOVA, * p<0.05, **p<0.01.

Lipids can be transferred from adipocytes to cancer cells (3). Pre-labelled primary mouse subcutaneous adipocytes with a fluorescent fatty acid analogue were co-cultured with mouse MET-1 cancer cells under normoxic and hypoxic conditions to investigate whether the increased lipolytic effect under hypoxia would increase the transfer of lipids to cancer cells (**Figure 4A**). In hypoxia, WT adipocytes had a significantly higher transfer of lipids towards cancer cells (**Figure 4B**) compared to HIF1α-deficient adipocytes. This is consistent with our data showing that cancer cell-induced lipolysis is blunted in the HIF1α-defiecient adipocytes, therefore the availability of lipids from these cells is reduced.

### Hypoxia causes perturbations in the human adipocyte metabolome

**Figure 5:**
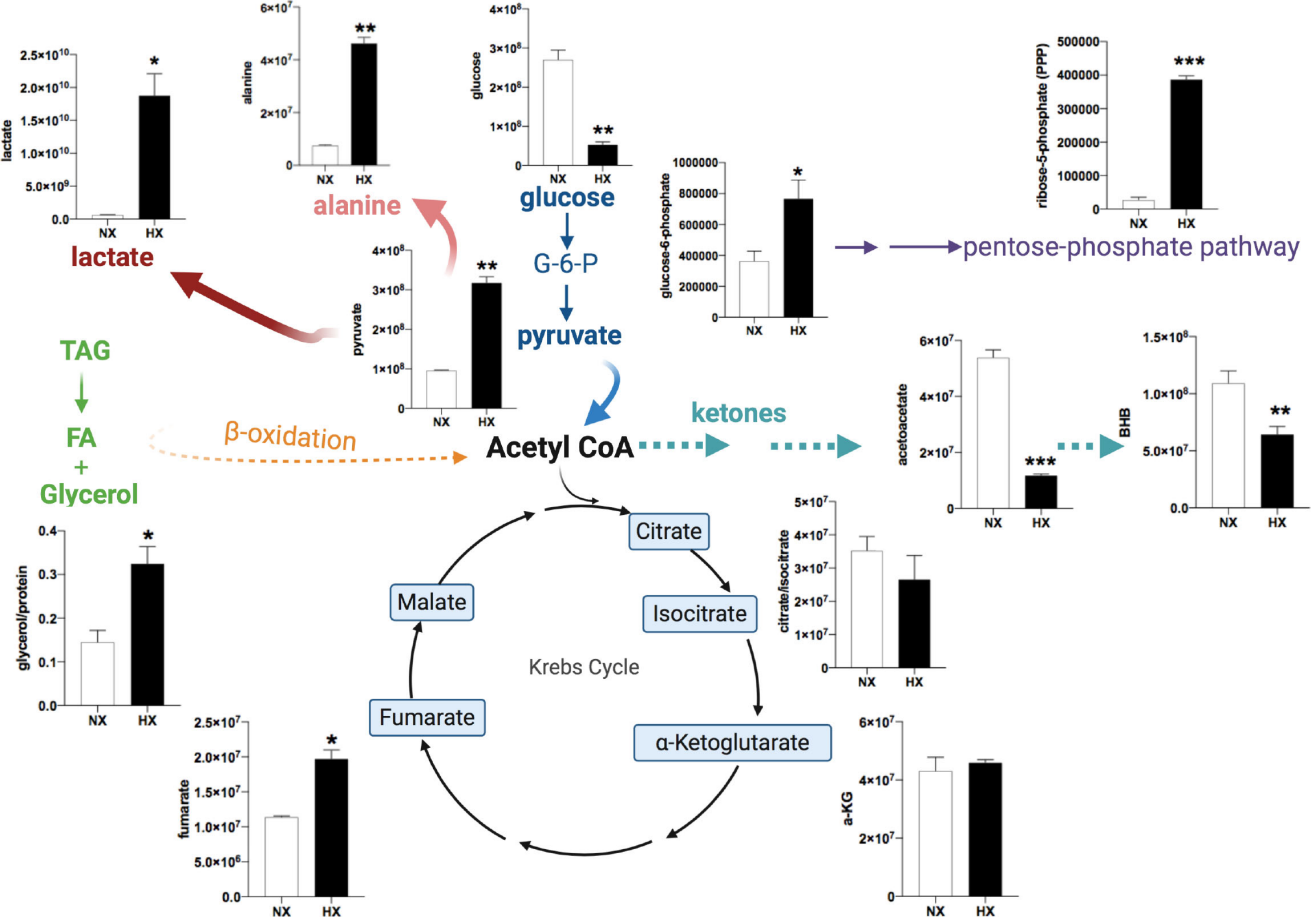
Human adipocyte metabolome is modulated in the presence of breast mammary cells under hypoxia. SGBS adipocytes were cultured with MCF-10A cells CM in normoxia (NX, 21% O2, white bars) or HX (0.5% O2, black bars) for 24h.Targeted LC/MS analysis in SGBS media (n=3/group). Representation of changes in energy metabolites, TCA cycle intermediates, pentose phosphate pathway (PPP) intermediates. Ordinate axes represent metabolite peak area. Values presented as mean+/- SEM. Significance by Students t-test *p<0.05, **p<0.01, ***p<0.001. Note: in Nx, adipocytes and CCM was prepared in Nx. In Hx, adipocytes and CCM in Hx.

Although lipids (fatty acids and glycerol) are the major metabolites released from adipocytes, we hypothesised that hypoxia-driven changes in other metabolites in adipocytes could provide energetic and biosynthetic substrates to neighbouring cells. The non-malignant mammary cell line, MCF-10A, was used to generate conditioned media. Hypoxia can induce the transformation of non-malignant cells and potentially initiate early stages of tumorigenesis (29). Here we investigated whether interaction with non-transformed mammary cells can alter adipocyte metabolism under hypoxic conditions. Human SGBS adipocytes were cultured with hypoxia-preconditioned MCF-10A (HxCM; 0.5% O2 for 24h) under 21% or 0.5% O2 for 24h. Medium from SGBS adipocytes was collected and analysed by LC-MS using a targeted metabolomics approach. As with the cancer cell lines, even non-tumorigenic mammary cells under hypoxic conditions increased glycerol release from adipocytes (**Figure 5**). Hypoxia per se in adipocytes induced changes in glucose/lactate/fumarate levels (**Supplemental Figures S3A–C**), but the effects were significantly more pronounced in the presence of MCF-10A CM (**Figure 5**). Analysis of metabolites in the media by LC-MS revealed that, in combination of hypoxia and CM, glucose levels were depleted in adipocytes whereas pyruvate and lactate (**Figure 5**) were dramatically increased confirming a hypoxia-driven glycolytic effect in adipocytes. Increased pyruvate abundance, most likely coupled with a lack of pyruvate entry into the oxidative TCA cycle (due to hypoxia) led to significant increases in alanine and fumarate, metabolites that are derived from pyruvate through transamination and carboxylation/reduction, respectively. Significant increase was also seen in ribose-5-phosphate levels (**Figure 5**) driven by elevated glucose-6-phosphate levels. In contrast, ketone bodies, acetoacetate and beta-hydroxybutarate (BHB) were significantly reduced during hypoxia. Hypoxia alone (without CM) did not affect BHB levels in adipocytes (**Supplemental Figure S3D**). Finally, proliferation of the MCF-10A cells was increased under hypoxic conditions when given the hypoxic-ACM from SGBS cells (**Supplemental Figure S4**) suggesting that these metabolic shifts in adipocytes can provide a proliferative advantage to non-malignant cells.

## Discussion

**Figure 6:**
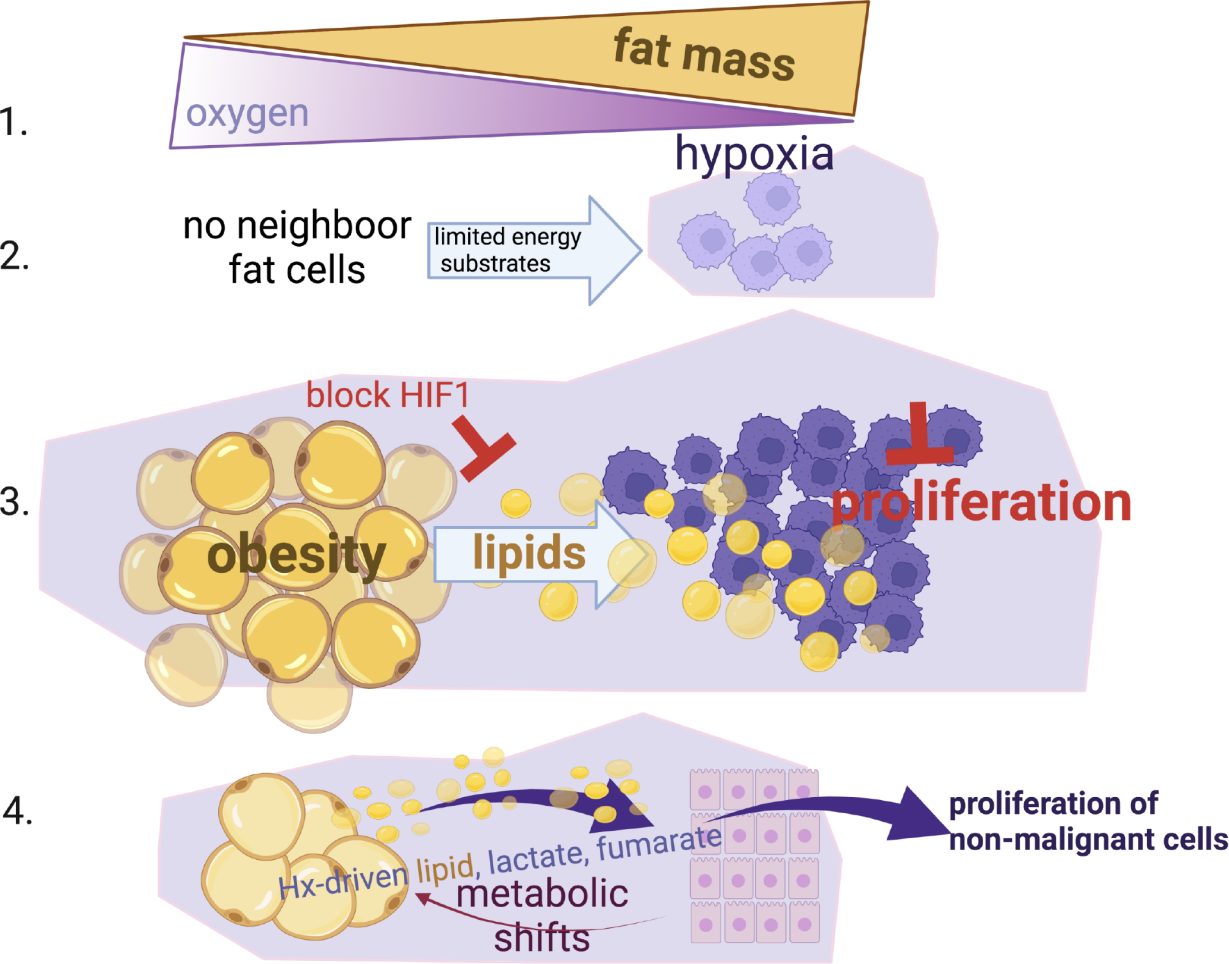
Summary Schematic. (1) Fat expansion in obesity shares a common pathogenic feature with the cancer micro-environment, reduction of local oxygen levels. (2) Hypoxia limits the proliferation of cancer cells especially when nutrients are not available from neighbour fat cells. (3) When cancer cells reside close to fat cells (i.e. breast or ovarian omental metastasis), they induce lipid release from fat cells, hijack and use these lipids to by-pass the hypoxic-inhibitory effect on proliferation in a HIF-1α dependent manner. (4) Non-malignant mammary cells when reside in hypoxic environments can also facilitate metabolic shifts in fat cells (increased lactate, fumarate, lipids) and increase their proliferation status. This suggests that an obesogenic hypoxic microenvironment could be potentially a driver of cancer growth.

Here we show that hypoxia, a common characteristic in the obese and tumour microenvironment, is key in the metabolic perturbation of adipocytes and their neighbouring cancer cells. Hypoxia drives excess supply of lipids to cancer cells, in a HIF-1a-dependent manner. We show that hypoxia also causes changes in metabolic pathways (glycolysis, ketosis, TCA intermediates, PPP) in adipocytes that allow normal breast epithelial cells to have a more proliferative phenotype that could potentially facilitate transformation and promote oncogenesis (summary **Figure 6**).

It is well appreciated that the stroma (including pre-adipocytes/adipocytes) is a critical determinant in tumour progression and metastasis (30, 31) and growing evidence shows that metabolic adaptation is a key factor in tumour progression (32). The effects of hypoxia in cancer cells are well established as metabolic adaptations driven by HIF-1α provide a selective advantage for cancer cells in the low oxygen environment (15, 33). To cope with hypoxia, cancer cells adapt towards increased glycolysis and glutaminolysis (34, 35). Enhanced HIF-1α activity in cancer cells has been shown to increase fatty acid uptake in response to hypoxia (15, 36). Hypoxia is also the primary trigger of fatty acid oxidation in cancer cells that reside close to adipose tissue (37).

Crucially, in order to understand the causal links between obesity and breast cancer we need to study the metabolic status of these cells in the conditions of oxidative deprivation in which these cells reside. The metabolic shifts in adipocytes residing close to cancer cells in the hypoxic microenvironment are less known. Hypoxia in obese adipose tissues affects the cellular/molecular processes in the adipose microenvironment by inhibiting adipocyte differentiation from preadipocytes, stimulating inflammatory responses of macrophages and triggering fibrosis (10). Recent findings show that the hypoxic obese adipose tissue would further elevate tumour hypoxia (37). To maintain their ATP levels under hypoxia, adipocytes depend on increased glycolysis. This metabolic switch coupled with the lipolytic drive, reported here, may exhaust adipocyte energetic reserves and could thereby lead to further impaired glucose tolerance, linked to cancer progression (38). Our data show that both breast and ovarian cancer cells under hypoxia enhance lipid release from adipocytes and these lipids are taken up by the cancer cells to facilitate proliferation under hypoxic conditions. This process is HIF-1α dependent as deletion of HIF1α in adipocytes blunts lipid release and access of lipids to cancer cells. Although the focus in our studies was the effect of lipids and adipose-derived metabolites on cancer cell proliferation, as hypoxia has a well-established inhibitory effect, this is a limitation of our study. For tumour progression, other cross-regulatory pathways in the tumour-adipose microenvironment (TAME), such as extracellular matrix (EMT) remodelling, maintenance of stemness, blood vessel formation, modulation of tumour metabolism, immune response, migration, invasion need to be considered.

An important question is whether hypoxia in the non-tumorigenic adipose/breast stroma could cause metabolic shifts and early stages of transformation. Indeed, hypoxia can lead to a cancer-like phenotype in immortalised (but untransformed) human breast epithelial cells (MCF-10A) and influence cells in the earliest stages of transformation (29). Supporting evidence also comes from adipocytes that can modify estrogen receptor (ER) gene expression through hypoxia and can promote EMT processes in breast cancer cells (39). This could implicate early metabolic changes of neighbouring adipocytes under hypoxia that in a vicious cycle could influence the fate of mammary epithelial cells towards a pro-malignant environment in breast tissue.

Our metabolic analysis on human adipocytes cultured with CM from normal mammary epithelial cells in hypoxia showed that a variety of metabolic circuits were modulated. Pyruvate excess was central to elevated (a) lactate and (b) alanine release and (c) accumulation of fumarate, most likely through the action of pyruvate carboxylase and reductive metabolism through mitochondrial malate dehydrogenase. Fumarate has been linked to mitochondrial stress in adipocytes of hyperglycaemic mice causing fumarate-dependent protein succination (40) that could lead to oncogenesis. Hypoxia-induced accumulation of oncometabolites can be amplified by the oncometabolites themselves as they can stabilize HIF1α *via* direct inhibition of the HIF-degradation enzymes HIF-prolyl hydroxylases (PHDs) (41–43). Abundance of adipose-derived lipids and other metabolic products could therefore provide a detrimental advantage towards oncogenesis. An interesting finding that warrants further investigation is that hypoxia reduced ketone bodies, the end-products of fatty acid metabolism. This is possibly due to enhanced TCA activity, so acetyl-coA availability towards ketone production was reduced. Although ketone bodies are mainly produced by the liver, adipocytes can also produce BHB (44). Ketone bodies are not a preferred energy substrate in cancer cells, as they can limit availability of glycolytic substrates and reduce proliferation (45), therefore we could speculate that increasing ketone body availability in the tumour microenvironment, we could create an unfavourable metabolic environment for the cancer cells. Ketogenic diets (KD) in preclinical models have shown to reduce cancer progression (46). Whether KD can affect adipocyte metabolism and influence substrate availability to cancer cells, especially in obesity-related cancers remains unknown. Although, our approach to detect metabolites secreted from the adipocytes reports robust changes expected under hypoxic conditions, in order to be able to extrapolate dynamic changes in metabolic activity in a specific pathway, an isotope tracing (i.e. 3C-pyruvate) future study would help us elucidate the flux in adipocytes. Similarly, our focus here was on the interactions of adipocytes with cancer cells, however, in future studies, to mimic the tumour micro-environmental conditions (oxygen tension, tissue dimensionality, heterogeneity), in a pathologically more relevant manner, 3D models should be tested (eg multicellular tumour spheroids with hypoxic core embedded in stroma).

Several studies highlighted that metabolic imbalance, hyperlipidaemia and hyperglycaemia, in obesity and T2D could be causal to breast cancer progression. Particularly T2D-derived adipocytes can be critical in TAME by directly providing lipids for cancer cell growth, proinflammatory cytokines or exosomes that facilitate tumour aggressiveness associated with EMT and cancer cell stemness (3, 47, 48). Here we show that the hypoxic microenvironment is a critical factor for this link. The hypoxia-driven metabolic perturbations in the adipocytes, for example the accumulation of oncometabolites, like fumarate coupled with hyper-glycemia found in obese-diabetic patients could influence phenotypic switches, like EMT, of normal mammary epithelial cells, and subsequent tumour initiation processes. Further hypoxic stress in adipocytes is predicted to fuel the cancer cells by provision of nutrients (lipids, glucose, amino acids) for their growth. Especially, lipid uptake for the biosynthesis of cancer cell membranes will facilitate cell integrity and growth, while generation of other lipid-derived biomolecules (such as steroid hormones, phospholipids, diacyglycerols etc) will further sustain cancer cell function (43). Indeed, blocking the uptake of FA by inhibition of fatty acid receptor CD36 has been shown to impair metastasis in human breast cancer-derived tumours (17).

In terms of therapy, a huge hurdle in cancer patients is antiangiogenic drugs resistance. Recently, a striking finding was that anti-VEGF-treated tumours grown in adipose experienced a high magnitude of hypoxia (37). This undesirable effect of anti-angiogenic therapies combined with the metabolic perturbations (especially accelerated lipolysis/glycolysis) we found in hypoxic adipocytes could potentially worsen further the outcome of the cancers that reside close to adipose tissues. It warrants further investigation whether targeting the HIF-pathway in cancers that hijack metabolic by-products from adipocytes would help identify more efficient therapies.

## Data availability statement

The raw data supporting the conclusions of this article will be made available by the authors, without undue reservation.

## Ethics statement

The studies involving human participants were reviewed and approved by Ethical approval (reference number 15/ES/0094) for the collection, storage and subsequent use of human adipose tissue was granted by The Human Tissue (Scotland) Act, 2006. The patients/participants provided their written informed consent to participate in this study. The animal study was reviewed and approved by Animal studies were performed under licensed approval in accordance with the U.K. Home Office Animals (Scientific Procedures) Act, 1986.

## Author contributions

RA performed experiments and analysed data. JW and AF performed and analysed the LC/MS data. KFR, CB, RHS, MW and JWP provided resources and/or contributed to data discussions. ZM conceived the study, designed/performed experiments, analysed data and wrote the manuscript which was reviewed by all authors.

## Acknowledgments

This work was supported by a Wellcome Trust ISSF3 and a British Heart Foundation REA2 (RE/13/3/30183) to ZM. Human adipose tissue collections were supported by the Chief Scientist Office (SCAF/17/02) to SR. JWP is funded by Wellcome Trust 101067/Z/13/Z, MRC Centre grant MR/N022556/1 and a CRUK programme grant C17950/A26783. Schematics were created with licenced BioRender.com. For the purpose of open access, the author has applied a CC-BY public copyright licence to any Author Accepted Manuscript version arising from this submission.

## Conflict of interest

The authors declare that the research was conducted in the absence of any commercial or financial relationships that could be construed as a potential conflict of interest.

## Publisher’s note

All claims expressed in this article are solely those of the authors and do not necessarily represent those of their affiliated organizations, or those of the publisher, the editors and the reviewers. Any product that may be evaluated in this article, or claim that may be made by its manufacturer, is not guaranteed or endorsed by the publisher.
