# Supplementary material for: Hypoxia-driven metabolic reprogramming of adipocytes fuels cancer cell proliferation
Source: Front Endocrinol (Lausanne). 2022 Oct 18;13:989523. doi: 10.3389/fendo.2022.989523 (PMC9623062; doi:10.3389/fendo.2022.989523)
Supplement: Supplementary file 1 [file Presentation_1.pdf]

Supplemental Figure 1

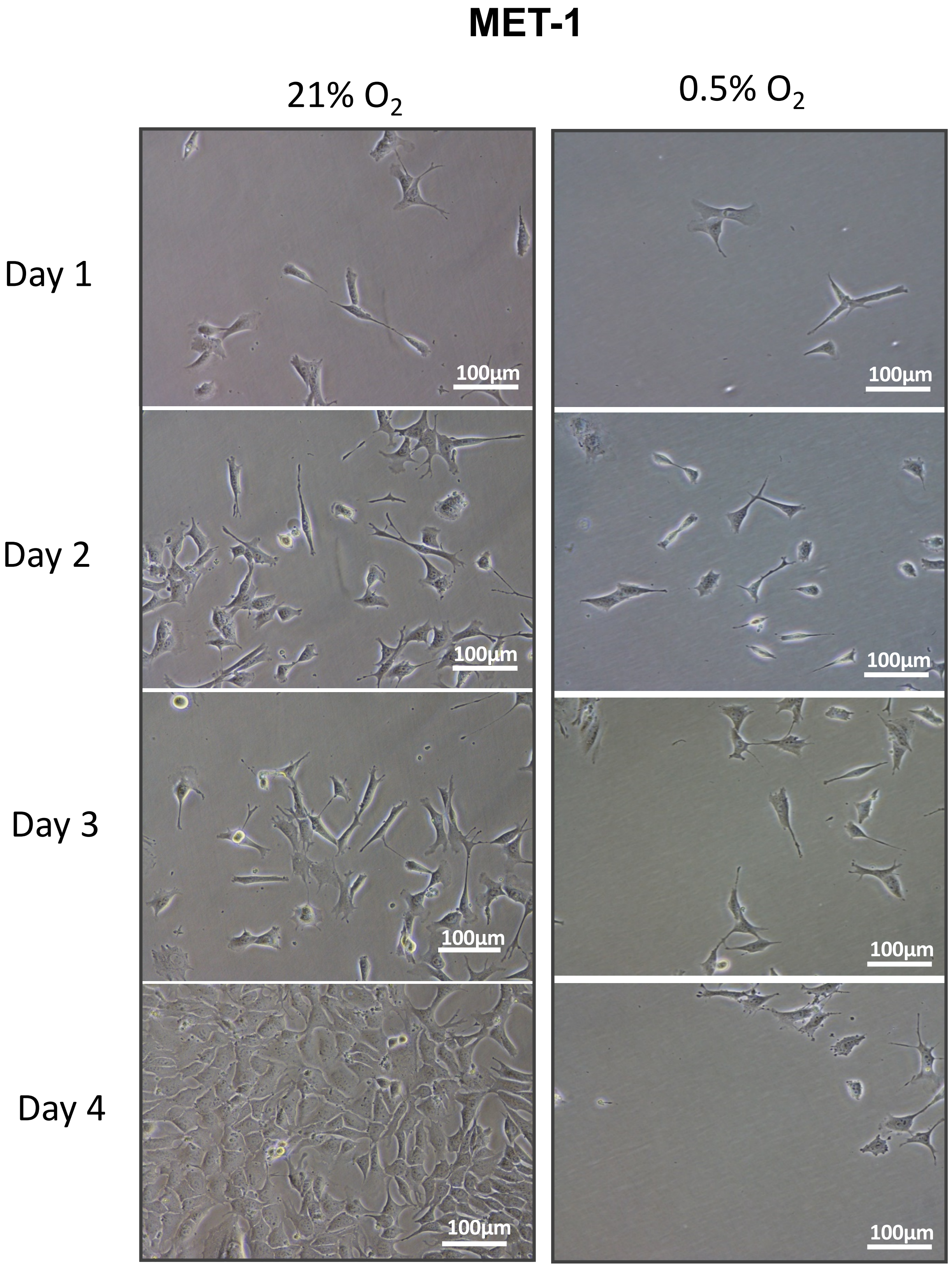

Supplemental Figure 1. Hypoxia inhibits growth of the mouse MET-1 cancer cells. $2x10^5$ cells were seeded and cultured for 4 days in normoxia (21% O_2) or hypoxia (0.5% O_2). Growth of cells was monitored at the indicated time points. Representative images of n=3/group**.** Scale bars=100µm

Supplemental Figure 2

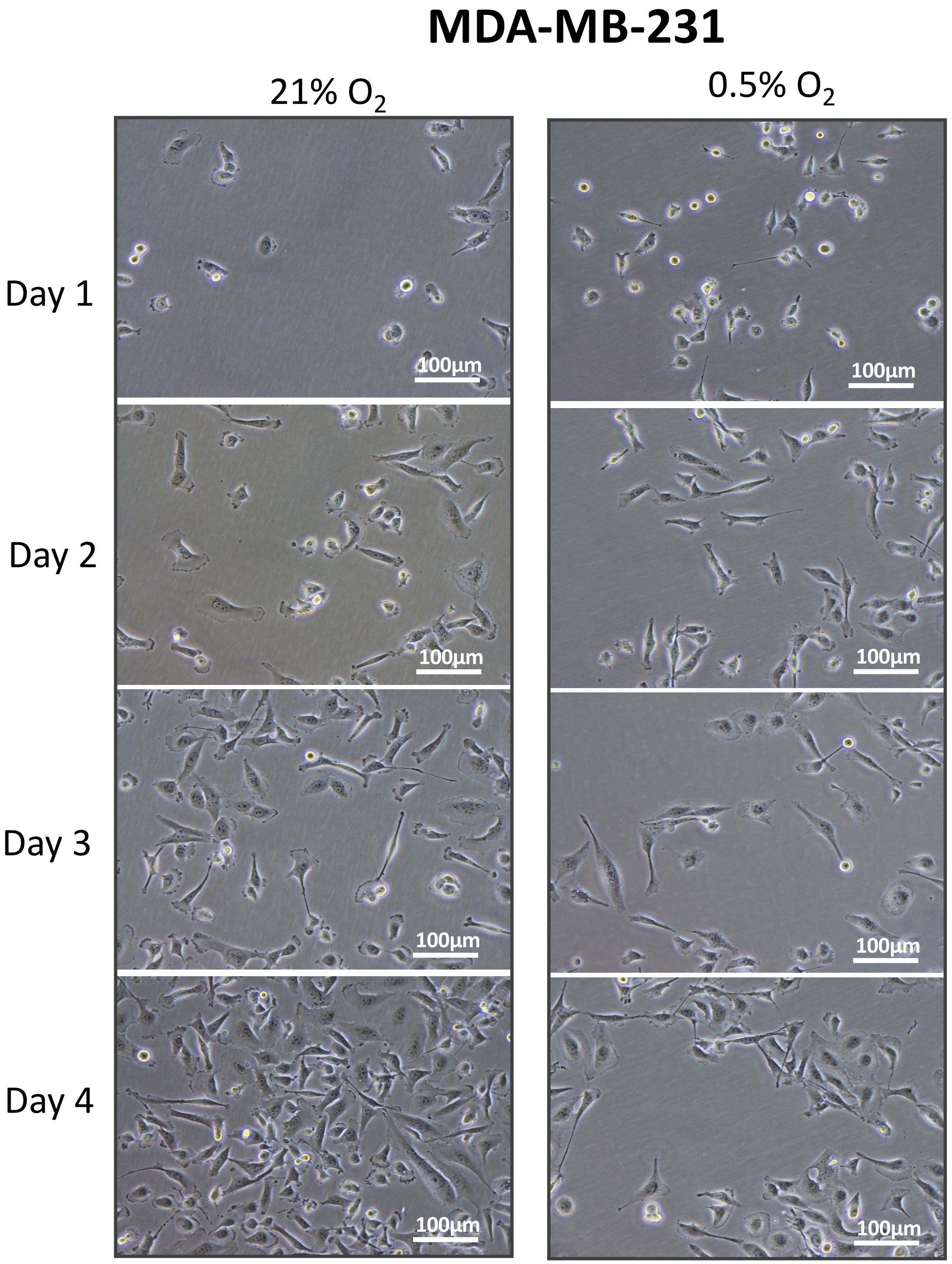

Supplemental Figure 2. Hypoxia inhibits growth of the human MDA-MB-231 cancer cells. $5x10^5$ cells were seeded and cultured for 4 days in normoxia (21% O_2) or hypoxia (0.5% O_2). Growth of cells was monitored at the indicated time points. Representative images of n=3/group. Scale bars=100µm

Supplemental Figure 3

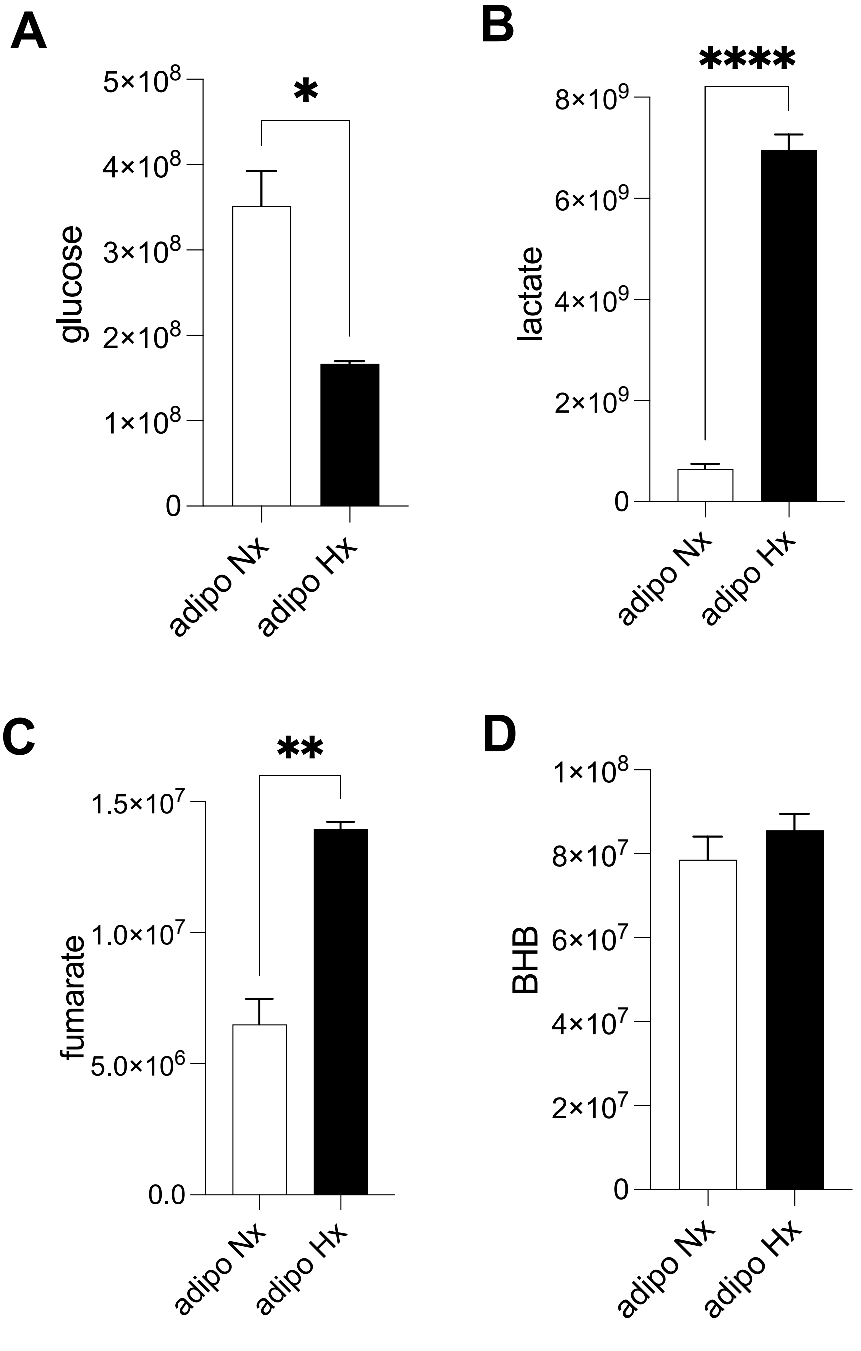

Supplemental Figure 3. Human adipocyte metabolite changes in hypoxia. cells under hypoxia. SGBS adipocytes were cultured in normoxia (NX, 21% O_2, white bars) or hypoxia (HX,0.5% O_2, black bars) for 24h.Targeted LC/MS analysis performed in SGBS media (n=3/group). Representation of changes in (A) glucose, (B) lactate, (C) TCA cycle intermediate fumarate and (D) the ketone body, β-hydroxybutyrate. Ordinate axes represent metabolite peak area. Values presented as mean+/- SEM. Significance by Students t-test *p<0.05, **p<0.01, ***p<0.001.

Supplemental Figure 4

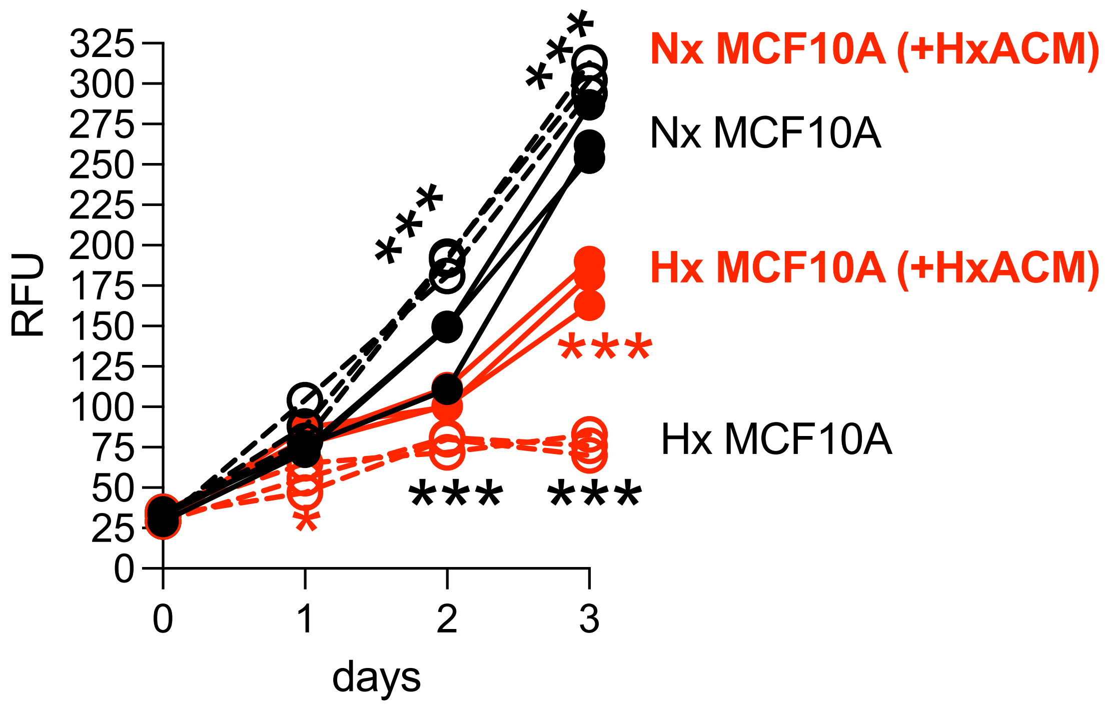

Supplemental Figure 4. Hypoxic-adipocyte conditioned media facilitates proliferation of non-malignant MCF10A cells in hypoxia. *In vitro* proliferation (RFU, relative fluorescence units) of the non-malignant human mammary epithelial cells, MCF10A, cultured in CM from hypoxic SGBS adipocytes for 3 days in normoxia (Nx=21% O_2) or hypoxia (Hx=0.5% O_2). N=3/group. Values are each replicate aligned (n=3/group). * p<0.05, ***p<0.001. Note, red stars indicate comparison under hypoxia between MCF10A and MCF10A+HxACM.
